# Isolation and Characterization of a Thermotolerant Ammonia-Oxidizing Bacterium *Nitrosomonas* sp. JPCCT2 from a Thermal Power Station

**DOI:** 10.1264/jsme2.ME13058

**Published:** 2013-11-19

**Authors:** Yoshikane Itoh, Keiko Sakagami, Yoshihito Uchino, Chanita Boonmak, Tetsuro Oriyama, Fuyumi Tojo, Mitsufumi Matsumoto, Masaaki Morikawa

**Affiliations:** 1 Division of Biosphere Science, Graduate School of Environmental Science, Hokkaido University, Kita 10 Nishi 5, Kita-ku, Sapporo 060–0810, Japan; 2 NITE Biological Resource Center (NBRC), National Institute of Technology and Evaluation (NITE), 2–5–8 Kazusakamatari, Kisarazu, Chiba 292–0818, Japan; 3 Wakamatsu Research Institute, Technology Development Center, Electric Power Development Co., Ltd., Yanagisaki, Wakamatsu, Kitakyushu, Fukuoka 808–0111, Japan

**Keywords:** *Nitrosomonas*, thermotolerant ammonia-oxidizing bacterium, activated sludge

## Abstract

A thermotolerant ammonia-oxidizing bacterium strain JPCCT2 was isolated from activated sludge in a thermal power station. Cells of JPCCT2 are short non-motile rods or ellipsoidal. Molecular phylogenetic analysis of 16S rRNA gene sequences demonstrated that JPCCT2 belongs to the genus *Nitrosomonas* with the highest similarity to *Nitrosomonas nitrosa* Nm90 (100%), *Nitrosomonas* sp. Nm148 (99.7%), and *Nitrosomonas communis* Nm2 (97.7%). However, G+C content of JPCCT2 DNA was 49.1 mol% and clearly different from *N. nitrosa* Nm90, 47.9%. JPCCT2 was capable of growing at temperatures up to 48°C, while *N. nitrosa* Nm90 and *N. communis* Nm2 could not grow at 42°C. Moreover, JPCCT2 grew similarly at concentrations of carbonate 0 and 5 gL^−1^. This is the first report that *Nitrosomonas* bacterium is capable of growing at temperatures higher than 37°C.

Chemolithoautotrophicammonia-oxidizing bacteria (AOB), which convert ammonium to nitrite, play an important role in the global cycling of nitrogen (19, 22). Isolation of AOB was first reported in 1890 (2, 28), and since then a considerable number of AOB within the Betaproteobacteria and Gammaproteobacteria have been obtained from various environments (6, 10, 23, 26, 29). In particular, members of the betaproteobacterial genera, *Nitrosomonas* and *Nitrosospira*, are considered as the most dominant AOB in activated sludge (4, 14, 15, 20). Most strains of *Nitrosomonas* and *Nitrosospira* preferably grow in a relatively narrow range of moderate temperatures between 25 and 30°C (3).

It has been recently recognized that geothermal environments are also favorable habitats for AOB and ammonia-oxidizing archaea (AOA) (31). There are several reports on isolation and characterization of thermophilic AOA (5, 18); however, AOB cultures are unstable at high temperatures and no successful isolation has been reported (13). Here, we report for the first time the isolation of a thermotolerant AOB from activated sludge in a wastewater treatment plant continuously operated at 37–45°C.

## Materials and Methods

### Isolation of and physiological characterization of JPCCT2

A JPCC (J-Power Culture Collection) T2 (water treatment tank) bacterium sample was isolated from activated sludge in a thermal power station of the Electric Power Development Co., Ltd (J-Power) (Fukuoka, Japan). Enrichment cultures were repeated several times at intervals of 7 days in modified Alexander (MA) medium containing, per liter, 2 g of (NH_4_)_2_SO_4_ (30 mM ammonium) as the sole source of nitrogen, 0.5 g NaHCO_3_ (6 mM carbonate) as the sole source of carbon, 0.5 g K_2_HPO_4_, 50 mg MgSO_4_·7H_2_O, 5 mg CaCl_2_·2H_2_O, 2 mg MnSO_4_·4H_2_O, 5 mg Fe-EDTA(III), 0.1 mg CuSO_4_·5H_2_O, 0.05 mg Na_2_MoO_4_·2H_2_O, 0.001 mg CoCl_2_·6H_2_O, 0.1 mg ZnSO_4_·7H_2_O, and 50 mM HEPES (pH 7.8) (30) at 28°C with rotary shaking at 130 rpm. MA solid medium containing 1% gellan gum in MA medium (25) was used for single colony isolation of AOB after sub-culturing for two months. Consumption of ammonium and production of nitrite was confirmed in every culture using the Ammonia-test (Wako, Osaka, Japan) and naphthylethylenedi-amine spectrophotometric analysis (8), respectively. Sucrose density gradient centrifugation was further applied for isolation of JPCCT2. Culture purity was confirmed by the non-growth test in LB medium (1% NaCl, 0.5% yeast extract, and 1% peptone, pH 7.2) and also by no multiple peaks at single base positions in the 16S rRNA gene sequence raw data (ABI3130; Applied Biosystems, Carlsbad, CA, USA) for JPCCT2.

JPCCT2 and three related *Nitrosomonas* strains, *N. nitrosa* Nm90, *N. europaea* IFO14298 (= NBRC14298, ATCC19718) and *N. communis* Nm2, were pre-grown for 3 days at 28°C in a rotary shaker (130 rpm) or standing (for Nm90). *N. europaea* IFO14298 was obtained from NBRC. *N. nitrosa* Nm90 and *N. communis* Nm2 were kind gifts from Dr. Andreas Pommerening-Röser (University of Hamburg). MA medium was used to culture *N. europaea* and *N. communis*, and Medium Ia or Ib was used for *N. nitrosa. N. nitrosa* was sensitive to strong aeration and was mostly grown under standing culture conditions. Medium Ia contained, per liter, 535 mg NH_4_Cl (10 mM ammonium) as the sole source of nitrogen, 54.4 mg KH_2_PO_4_, 74.4 mg KCl, 147 mg CaCl_2_·2H_2_O, 49.3 mg MgSO_4_·7H_2_O, 1 ml trace element solution, and 1 ml of 0.05% Cresol Red solution, and pH was maintained at 7.8 by 5 g CaCO_3_ (Medium Ib) or appropriate addition of 10% NaHCO_3_. Trace elements solution contained, per liter, 3.5 mM FeSO_4_, 0.8 mM H_3_BO_3_, 0.15 mM ZnSO_4_, 0.1 mM CuSO_4_, 0.03 mM (NH_4_) _6_Mo_7_O_24_, 0.02 mM MnSO_4_, and 0.025 N HCl. After washing with fresh medium, the cells of each strain were inoculated at the appropriate OD_600_ into each medium for growth tests under different conditions of temperature (28, 37, 43, 45 and 48°C), ammonium (7.5, 11.25, 15.0 and 30.0 mM), sodium bicarbonate (0 or 5.0 g L^−1^), and sodium chloride (100, 300 and 500 mM). Temperature for cultivation was generally 28°C unless otherwise denoted.

### Biochemical characterization

G+C content (mol%) of DNA was directly determined by complete hydrolysis of the genomic DNA followed by quantification of each nucleoside by HPLC according to the protocols for the DNA GC Kit (Seikagaku Biobusiness, City, Country). The score was calculated as the average of three independent experiments. Genomic DNA was purified according to the protocol for the GenElute Bacterial Genomic Kit (Sigma-Aldrich, St. Louis, MO, USA).

Respiratory quinones were extracted from the cells in the stationary phase of JPCCT2 culture, 3–7 days, according to the protocol of (16) and analyzed with an LCMS-8030 spectrometer (Shimadzu, Kyoto, Japan).

Fatty acid methyl esters were prepared according to the standard protocol described in the MIDI microbial identification system (Microbial ID; Agilent Technologies, City, Country) and analyzed by GC-MS (GC system model 6890 and MSD model 5973; Agilent Technologies) using Sherlock MIDI software (version 4.0) and the TSBA database (version 4.0).

### 16S rRNA gene sequence and phylogenetic tree analyses

Approximately 1,500 bp of the 16S rRNA gene from JPCCT2 was amplified by PCR using a set of 8–27 forward (5′-AGA GTT TCC TGG CTC AG-3′) and 1510–1492 reverse (5′-GGC TAC CTT GTT ACG ACT T-3′) primers.

The original sequence (1,460 bp) excluding the primer sequences was registered under DDBJ/EMBL/GenBank AB610420. The sequence was compared to the NCBI database (http://www.ncbi.nlm.nih.gov) using the BLAST program (http://blast.ncbi.nlm.nih.gov/Blast.cgi) (10). The 16S rRNA gene sequences from ten strains in *Nitrosomonas*, *Nitrosococcus mobilis* Nm93, and *Nitropina gracilis* 3/211^T^ (as an outgroup strain) were retrieved from the NCBI database. Phylogenetic trees were constructed by the neighbor-joining (NJ) method. The NJ tree was constructed from evolutionary distance data corrected by two-parameter transformation of (11), using the neighbor-joining method of MEGA version 5 (17, 24).

### Accession numbers of the 16S rRNA sequence data obtained from GenBank

AY123795 (*Nitrosomonas eutropha* Nm57), AB070982 (*N. europaea* ATCC25978^T^), AJ298731 (*Nitrosomonas halophila* Nm1), AF037105 (*Nitrosomonas mobilis* Nm93), AB610420 (*Nitrosomonas* sp. JPCCT2), FR828477 (*N. nitrosa* Nm90), AJ298732 (*N. communis* Nm2), AF272414 (*Nitrosomonas ureae* Nm10), AJ298736 (*Nitrosomonas oligotropha* Nm45), AF272418 (*Nitrosomonas marina* Nm22), AJ298734 (*Nitrosomonas aestuarii* Nm36), AF272423 (*Nitrosomonas cryotolerans* Nm55), and FR865038 (*Nitrospina gracilis* 3/211^T^).

## Results and Discussion

### Molecular characteristics

**Fig. 1:**
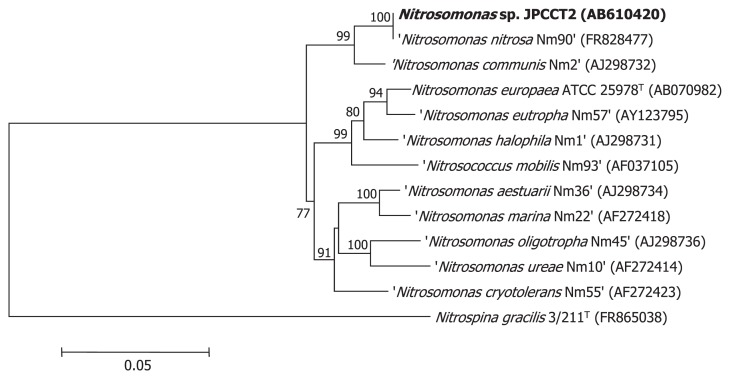
Phylogenetic trees based on the 16S rRNA gene sequence were constructed by the neighbor-joining method (NJ), showing the positions of *Nitrosomonas* sp. JPCCT2 and other related strains. Accession numbers of each gene sequence are shown in parentheses. Non-validated species name is denoted in half quotation marks. Numbers indicate percentages of bootstrap sampling, derived from 1,000 samples. *Nitrospina gracilis* 3/211^T^ was used as an outgroup strain.

An ammonia-oxidizing bacterium, designated JPCCT2 below, was isolated from activated sludge samples through enrichment cultures using ammonium and bicarbonate as sole nitrogen and carbon sources, respectively. The 16S rRNA gene of JPCCT2 showed significant sequence identity to species in the genus *Nitrosomonas: Nitrosomonas nitrosa* Nm90, 100% (1,460 bp); *Nitrosomonas* sp. Nm148, 99.7% (1,460 bp); *Nitrosomonas* sp. Nm41, 98.2% (1,460 bp); *Nitrosomonas* sp. Nm58, 97.8% (1,460 bp); *Nitrosomonas communis* Nm2, 97.7% (1,460 bp); *Nitrosomonas* sp. Nm33, 97.7% (1,453 bp) and *Nitrosomonas europaea* IFO14298 or ATCC25978, 92.8% (1,460 bp). The phylogenetic trees indicated that JPCCT2 was closely related to *N. nitrosa* and *N. communis* cluster (Fig. 1). G+C content of JPCCT2 DNA was determined as 49.1 mol%, different from other *Nitrosomonas* bacteria, including *N. nitrosa* Nm90 (47.9%) (Table 1).

### Chemotaxonomic properties

Ubiquinone-8 was the sole detectable respiratory quinone in JPCCT2. Ubiquinone-8 is a common form of ubiquinones among most Gram-negative bacteria, including *Nitrosomonas* bacteria (7). It was also found that JPCCT2 possessed a simple fatty acid composition mainly composed of C16:0 (42.8%) and C16:1ω7c (53.3%). It might be worth noting that fatty acids in *N. europaea* are C16:0 (25.0%), C16:1ω9c (61.6%), and C16:1ω9c (13.1%), while psychrotrophic *Nitrosomonas* sp. 4W30 are C16:0 (17 and 45%) and C16:1 (73 and 45%) at 5°C and 25°C, respectively (9, 21).

### Physiological and morphological characteristics

Table 1 summarizes the comparative characteristics of JPCCT2, *N. nitrosa* Nm90, *N. communis* Nm2, *N. europaea* IFO14298 and ATCC25978^T^. JPCCT2 cells were non-motile short rods or ellipsoidal with round ends whose size is relatively smaller than other *Nitrosomonas* bacteria. JPCCT2 showed moderate thermotolerance and grew at temperatures up to 48°C. In contrast to JPCCT2, *N. communis* and *N. nitrosa* were rather thermolabile when compared with other strains and could only slightly grow at 37°C. It is worth noting that the wastewater treatment tank T2, from which JPCCT2 was isolated, was continuously operated under 37–45°C conditions. More interestingly, the addition of sodium bicarbonate did not significantly affect the growth of strain JPCCT2 in the range of 0–5 g L^−1^, which also shows clear difference from *N. nitrosa* Nm90. Five grams per liter of sodium bicarbonate clearly inhibited the growth of *N. communis* Nm2 and led to cell lysis, and *N. europaea* IFO14298 grew normally at 5 g L^−1^ but could not grow without carbonate in the medium. *N. nitrosa* Nm90 could grow under neither of these extreme carbonate conditions. In contrast to these three strains, JPCCT2 grew similarly at both 0 and 5 g L^−1^ sodium bicarbonate at 28°C. pHs of the media were 4.7 and 8.3 with 0 g L^−1^ and 5 g L^−1^ sodium bicarbonate, respectively. This indicates that JPCCT2 and Nm2 could grow using a trace amount of naturally dissolved atmospheric carbon dioxide (mostly in the non-dissociated H_2_CO_3_ form at pH 4.7) in the medium with no addition of sodium bicarbonate. JPCCT2 utilized urea as a source of nitrogen and showed the maximum growth of 0.14 OD_600_ after 3 days’ cultivation at 28°C upon addition of 10 mM urea.

In conclusion, although the 16S rRNA gene sequence was very close to known *Nitrosomonas* species, the characteristics of G+C content, chemotaxonomy and physiological uniqueness, including thermotolerance and carbonate requirements, indicate that JPCCT2 might be a novel species in the genus *Nitrosomonas*.

### Description of *Nitrosomonas* sp. JPCCT2

Strict aerobe. Cells are Gram-negative rather small short rods or ellipsoidal with rounded ends, 0.5–0.7 μm wide and 0.9–1.6 μm long and exist mostly as singles. Motility is not observed. Cells pellets are slightly reddish in color. G+C content of the DNA is 49.1 mol%. Quinone type is ubiquinone-8. Utilize both ammonium and urea as sole nitrogen sources. Optimum (NH_4_)_2_SO_4_ concentration for growth is between 11.25 and 15.0 mM and additional 10 mM urea further stimulated growth. Optimum growth pH is between 7.5 and 8.0. Grew similarly at 0 and 5 g L^−1^ sodium bicarbonate. The range of growth temperature is wide, 28–48°C. JPCCT2 has been registered in culture collections (= JCM17640, = NBRC108559).

## Figures and Tables

**Table 1:** Major characteristics of JPCCT2, *N. nitrosa* Nm90, *N. communis* Nm2, *N. europaea* IFO14298, and *N. europaea* ATCC25978^T^. Growth was denoted by OD_600_ after 3 days and (1 day) for JPCCT2^T^, Nm2^T^, IFO14298, and 5 days and (3 days) for Nm90^T^. NG, <0.003 (no growth), ND, not determined, NA, not available. Initial OD_600_ of culture after inoculation was 0.01 for JPCCT2, Nm2, IFO14298, and 0.002 for Nm90.

Characteristics	*Nitrosomonas* sp. JPCCT2	*N. nitrosa* Nm90	*N. communis* Nm2	*N. europaea* IFO14298	*N. europaea* ATCC25978^T^
Source of strain	Activated sludge in a thermal power station	Industrial Sewage	Soil	Soil	Soil
Cell morphology	Short rods or ellipsoidal with round ends	Spheres or short rods with round ends	Short rods or ellipsoidal with round ends	Short rods or ellipsoidal with round ends or point ends	
Cell dimensions (μm)	0.5–0.7 × 0.9–1.6	1.3–1.5 × 1.4–2.2	1.0–1.4 × 1.7–2.2	0.8–1.1 × 1.0–1.7	
DNA G + C content (mol%)	49.1		46.1	50.4	
		47.9 (12)	45.8 (12)	50.7 (1)	50.5 (27)
Growth dependence on temperature					
28°C	0.078 [0.046]	0.026 [0.008]	0.084 [0.055]	0.100 [0.098]	NA
37°C	0.071 [0.059]	0.006 [0.006]	NG [0.033]	0.089 [0.090]	NA
42°C	0.063 [0.060]	NG	NG	0.034 [0.030]	NA
45°C	0.045 [0.043]	ND	ND	NG	NA
48°C	0.031 [NG]	ND	ND	ND	NA
Growth dependence on sodium bicarbonate					
0 g L^−1^ (pH 4.7)	0.062 [0.024]	NG	0.057 [0.018]	NG	NA
0.5 g L^−1^ (pH 7.8)	0.078 [0.046]	0.026 [0.008]	0.084 [0.055]	0.100 [0.098]	NA
5.0 g L^−1^ (pH 8.3)	0.079 [0.027]	NG	NG	0.077 [0.026]	NA
Use of urea	+	+ (12)	−	−	− (3)
Maximum tolerance to NaCl (mM)	300	300	300	500	500 (3)
